# Healing hearts: mind-body therapy for mothers after stillbirth’s silent grief

**DOI:** 10.3389/fpsyt.2025.1534616

**Published:** 2025-02-28

**Authors:** Vered Bar, Tamar Hermesh, Piki Reshef, Shoshy Hermetz, Nimrod Hertz-Palmor, Doron Gothelf, Mariela Mosheva

**Affiliations:** ^1^ Chava Center, Reproductive Psychiatry, Department of Psychiatry, Sheba Medical Center, Tel Hashomer, Israel; ^2^ The Child Psychiatry Division, Edmond and Lily Safra Children’s Hospital, Sheba Medical Center, Tel Hashomer, Israel; ^3^ Department of Obstetrics and Gynecology, Sheba Medical Center, Tel Hashomer, Israel; ^4^ MRC Cognition and Brain Sciences Unit, University of Cambridge, Cambridge, United Kingdom; ^5^ The Faculty of Medical & Health Sciences, Tel Aviv University, Tel Aviv, Israel; ^6^ Sagol School of Neuroscience, Tel Aviv University, Tel Aviv, Israel

**Keywords:** stillbirth, psychotherapy, depression, anxiety, post-traumatic stress disorders, suicide

## Abstract

**Introduction:**

Approximately 0.75% of pregnancies end with stillbirth, often leading to depression, anxiety, posttraumatic stress symptoms and suicidality. Knowledge regarding effective treatment options is lacking. In this retrospective cohort study we present a mind-body group therapy treatment protocol that was adapted for women and their partners who suffered stillbirth and report on its clinical effectiveness. Additionally, we identified demographic and clinical factors that were associated with clinical response.

**Methods:**

Sixty-one women who coped with stillbirth were enrolled to a mind-body group therapy. Questionnaires assessing symptoms of depression, anxiety, and post-traumatic stress were administered to the women before and after the group intervention.

**Results:**

As expected, we found high rates of depression, state and trait anxiety and post-traumatic symptoms in our cohort before mind-body group therapy. At completion of treatment, the symptoms of depression, state anxiety, post-traumatic stress and suicidality significantly decreased. Improvement in symptoms of depression and post-trauma at follow-up was positively associated with severity of symptoms at baseline and with antidepressants treatment, and negatively associated with the number of children. Time since stillbirth was positively associated with the degree of improvement in posttraumatic symptoms only.

**Conclusions:**

Our findings suggest that mind-body group therapy may be associated with improvements in depression, post-traumatic stress symptoms, state anxiety, and suicidal ideation in women following stillbirth. Further research, including a control group is crucial for understanding of effective tools to treat this at-risk population.

## Introduction

Still birth (SB) is defined as the delivery of a fetus with no signs of life at 20 or more weeks of gestation (1). More than 2.6 million SB cases occur globally each year (2). SB is an emotionally traumatic event that is compounded by disenfranchised grief, stigma and relationship stress and is often associated with symptoms of adjustment disorder, major depression, anxiety, and post-traumatic stress disorder (PTSD) (3). Women who have experienced SB have a higher rate of attempted and completed suicide compared to women who have delivered a live baby (4).

Most studies exploring mental outcomes following pregnancy loss (PL) have focused on grief response in a mixed cohort, such as women experiencing early and late pregnancy loss, neonatal loss, and recurrent miscarriages. In one study (3), levels of depressive and post-traumatic stress symptoms (PTSS) were found to correlate with the gestational week. Few studies have examined symptoms of major depressive disorder (MDD) and have shown that women who have experienced PL have higher rate of experiencing MDD compared to women who delivered a live baby (5, 6). Women who experienced PL have also been shown to have high rates of PTSS, which decline over time (7, 8). A study examining Israeli women following PL found high rates of PTSD (33.3%) and MDD (29.4%) and a high rate of comorbidity (9).

Many women who have experienced PL go on to conceive again within one year (10). The negative psychological impact of PL may linger and affect the course and outcome of the subsequent pregnancy (11, 12) including attachment to the following pregnancy and child (11, 13, 14).

Surprisingly, there are few empirical studies on how to best treat the negative psychological impact of SB. Several meta-analyses in recent years have pointed out the paucity of research in this area (13, 15, 16). Some studies have suggested that physical activity may provide mental health benefit after PL but have not suggested an intervention protocol (17). Other studies have examined interpersonal interventions, short-term online intervention, individual counseling by phone, couple and mindfulness-based interventions and grief counseling impact on grief, depression, PTSD, and anxiety symptoms (16–23). Most of these studies have examined mixed populations of early and late pregnancy loss and included small samples.

Mind-body therapy is a holistic therapeutic approach that combines techniques aimed at enhancing the connection between mental and physical health to promote overall well-being. It includes practices such as relaxation techniques, mindfulness, cognitive behavioral strategies, and psychoeducation to reduce stress, improve emotional resilience, and alleviate psychological or physical symptoms (24). It has been shown to benefit patients suffering from a variety of medical conditions in a various fields of medicine including oncology (25), neurology (26), pediatrics (27), mental health (28), and women undergoing *in vitro* fertilization (IVF) treatments (29, 30). In our study, participants were treated using a mind body group therapy (MBGT) protocol adapted for women who experienced SB. This MBGT protocol was originally developed for treating women facing infertility (31). However, to our knowledge, no studies have examined the feasibility of MBGT protocol nor its effectiveness for women after SB.

The primary objective of this study was to assess the effectiveness of MBGT intervention in alleviating symptoms of depression, anxiety, and PTSS as well as suicidal ideation in women who experienced SB. We hypothesized that women who underwent MBGT would show significant improvement in these symptoms. We further aimed to explore the demographic and clinical factors associated with clinical improvement following MBGT.

## Materials and methods

### Participants

Women included in MBGT were recruited from the Reproductive Psychiatry Center at Sheba Medical Center, a tertiary hospital located in the center of Israel. The Center receives approximately 100 new referrals each month, with 20-25% of these referrals involving women who have experienced stillbirth. Each woman underwent a psychiatric evaluation and was offered a range of personalized treatments, including dedicated groups for women after stillbirth. The study design involved a retrospective analysis using clinical questionnaires, along with demographic and clinical data. Women who agreed to participate in MBGT, were asked to complete these questionnaires both before entering MBGT (baseline) and following the final session of the eight-week intervention (follow-up).

A total of 72 women who experienced SB participated in MBGT between January 2018 and December 2021. Of these, 63 women completed questionnaires at both time points (baseline and follow up). We included only those who completed the questionnaires at both time points in our analysis. Exclusion criteria included (1) having more than eight children and (2) prior diagnosis of major psychiatric illness including schizophrenia, schizoaffective disorder or bipolar disorder. Based on these criteria, two women were excluded from the analysis, resulting in a final sample of 61 women for data analysis.

Sociodemographic characteristics, including age, marital status, educational attainment, number of children, past pregnancy outcomes, past or current psychiatric diagnosis, and current treatment with psychiatric medications, were assessed upon enrollment using self-report questionnaires. The questionnaires were part of the routine assessment of patients. The data were coded anonymously before the analysis. The Sheba Medical Center Institutional Review Board approved the study and the need for informed consent was waived due to the retrospective nature of the study.

### Intervention

In this study, we focused on the efficacy of MBGT in alleviating psychiatric symptoms in women after SB. This group intervention is an adaptation of a MBGT treatment protocol developed at Massachusetts General Hospital in Harvard, Boston, MA (31). The MBGT for women after SB teaches techniques that elicit a relaxation response, combined with additional resilience enhancing components such as cognitive behavioral therapy, behavioral activation, and psychoeducation. The group consists of eight sessions, two hours each. Partners are invited to the first session with the women. This session is dedicated to sharing the loss experience and is followed by psychoeducation and expectation management. Each of the subsequent six sessions starts with women sharing their experiences and feelings. Each session focuses on a different topic (disenfranchised grief, memory creation, relationship with the partner, family and community), followed by presenting tools to enhance resilience and practicing them. Each session ends with homework assignments and practicing relaxation together. The last session explores accomplished goals and thoughts about the next pregnancy. The partners are invited to a session for partners only. This session is dedicated to allowing the partners to share their difficulties along with psychoeducation.

### Outcome measures

Depression was assessed using the Edinburgh Post-natal Depression Scale (EPDS) (32, 33). The EPDS is a 10-item questionnaire assessing symptoms of depression during the last week. In the EPDS, each item is scored on a 4-point scale (range 0-3) and produces a total score ranging from 0 to 30, with scores of 10 or greater indicating possible depression. We used a cutoff score of 13 to minimize false positives (34). Item 10 of the EPDS examines suicidal ideation and its intensity. The EPDS showed good internal reliability (baseline Cronbach’s Alpha = .82), to sufficient internal reliability (follow up Cronbach’s Alpha = .77).

Anxiety was assessed using the State-Trait Anxiety Inventory (STAI) (35, 36). The STAI questionnaire is composed of 20 items for assessing trait anxiety (T-STAI) and 20 for assessing state anxiety (S-STAI). S-STAI evaluates the current state of anxiety. The T-STAI evaluates relatively stable aspects of anxiety proneness. Each item is scored on a 4-point scale (range 1-4). A cutoff score of 31 to 45 was used to determine moderate anxiety. Scores of 46 or higher were considered severe anxiety. The S-STAI showed excellent internal reliability (baseline Cronbach’s Alpha = .94, follow-up Cronbach’s Alpha = .93). T-STAI showed excellent internal reliability (baseline Cronbach’s Alpha = .89, follow up Cronbach’s Alpha = .84).

Post-traumatic stress symptoms were assessed using the PTSD check list for DSM-5 (PCL-5). The PCL-5 is a 20-item questionnaire that measures PTSS in the past month (37). Each item is scored on a 5-point scale (range, 0-4). A cutoff score of 38 was used to determine probable PTSD diagnosis (38). PCL-5 showed excellent internal reliability (baseline Cronbach’s Alpha = .93, follow up Cronbach’s Alpha = .92).

### Statistical analysis

Analyses were conducted using IBM’s Statistical Package for the Social Sciences (SPSS) Version 25. We used descriptive statistics to display the sociodemographic characteristics and clinical measures in the cohort. Paired t-tests were used to compare depression, PTSD and anxiety scores before and following intervention (baseline and follow up, respectively). We calculated effect sizes using Cohen’s standardized differences (Cohen’s *d*) to assess clinical significance in addition to statistical significance. Cohen’s *d* values of 0.2, 0.5, and 0.8 represent small, medium, and large effects, respectively. We dichotomized depression, PTSD and anxiety scores based on their cutoff scores, and used McNemar’s test to compare rates of above-cutoff scores at each time point. When our data did not meet the assumptions of the chi square test, we calculated *p* value using the binomial distribution.

Exploratory Pearson or Spearman’s correlation coefficients were calculated to assess the associations between our sample sociodemographic and clinical characteristics and the degree of improvement in symptoms of depression, PTSS and anxiety. We included in the exploratory correlation analysis only continuous or dichotomous variables. For continuous variables we used Pearson’s correlations and for dichotomous variables we used Spearman correlations. Family status was not included in the exploratory analysis as more than 90% of our cohort were married/in a relationship. Statistically significant correlations were further analyzed by multiple linear regressions. Separate models were conducted for each symptom cluster. Pre- to post-treatment differences in depression, PTSS and anxiety scores were defined as the dependent variables in each analysis. To control for multicollinearity, we detrended independent variables from one another and introduced their residuals to the model (39). This method does not affect the model’s general predictability but increases statistical power for each predictor, by accounting only for the portion of its variance which is not shared with other independent variables. The independent variables that were included after detrending were: baseline score for the specific questionnaire (continuous), treatment with antidepressants at baseline (binary), time since loss (measured in months), number of children (continuous). For all the analyses we used the standard α <.05 chance for a type I error.

## Results

### Sociodemographic and clinical sample characteristics

A total of 61 women (mean age 34.03 ± 5.27) were included in the analysis. Participants’ baseline characteristics are summarized in (**Table 1**). The mean gestational week of SB (SD) was 29.80 (6.00). A mean of 1.79 months had passed between SB and MBGT starting date. Thirty of the participants (49.2%) had at least one child. Ten women (16.4%) had an existing psychiatric diagnosis prior to SB: depressive disorders (n = 5) and anxiety disorders (n = 8). Two of these women were previously diagnosed with comorbid anxiety disorder and depressive disorder. One of these women was diagnosed with comorbid depression, obsessive compulsive disorder, and attention deficit/hyperactivity disorder. Nine women received pharmacological treatment before joining MBGT (all were treated with serotonin selective reuptake inhibitors (SSRI), and four of them also received benzodiazepines augmentation). One patient was treated with methylphenidate. Five women (8.2%) had IVF to conceive the pregnancy that was lost. Twenty-six of the women (42.6%) underwent pregnancy termination procedure.

**Table 1:** Sociodemographic and clinical characteristics of study sample.

Characteristic	Mean ± SD (range)	N (%)
Age	34.03±5.27 (23-48)	
Family Status Single Married/in a relationship Divorced		3 (4.9) 55 (90.1) 3 (4.9)
Place of birth Israel Other		55 (90.1) 6 (9.8)
Level of education Academic education (total) High school Above high school Missing		54 (88.5) 4 (6.5) 2 (3.3) 1 (1.6)
Chronic medical condition		14 (23)
Children (yes)		30 (49.2)
Number of living children	0.79±0.93 (0-3)	
Number of deliveries (including SB)	1.74±0.85 (1-4)	
Number of pregnancies	2.52±1.64 (1-8)	
Week of loss	29.8±6 (17-40)	
In-vitro fertilization		5 (8.2)
Termination procedure		26 (42.6)
Psychiatric history		10 (16.4)
Treatment with antidepressants medication following SB		9 (14.8)
Time since SB (months)	1.79±1.9 (0-9)	

### Intervention outcome

Women after SB participated in MBGT and showed significant improvement in symptoms of depression, PTSD, anxiety, and suicidal ideation.

**Figure 1:**
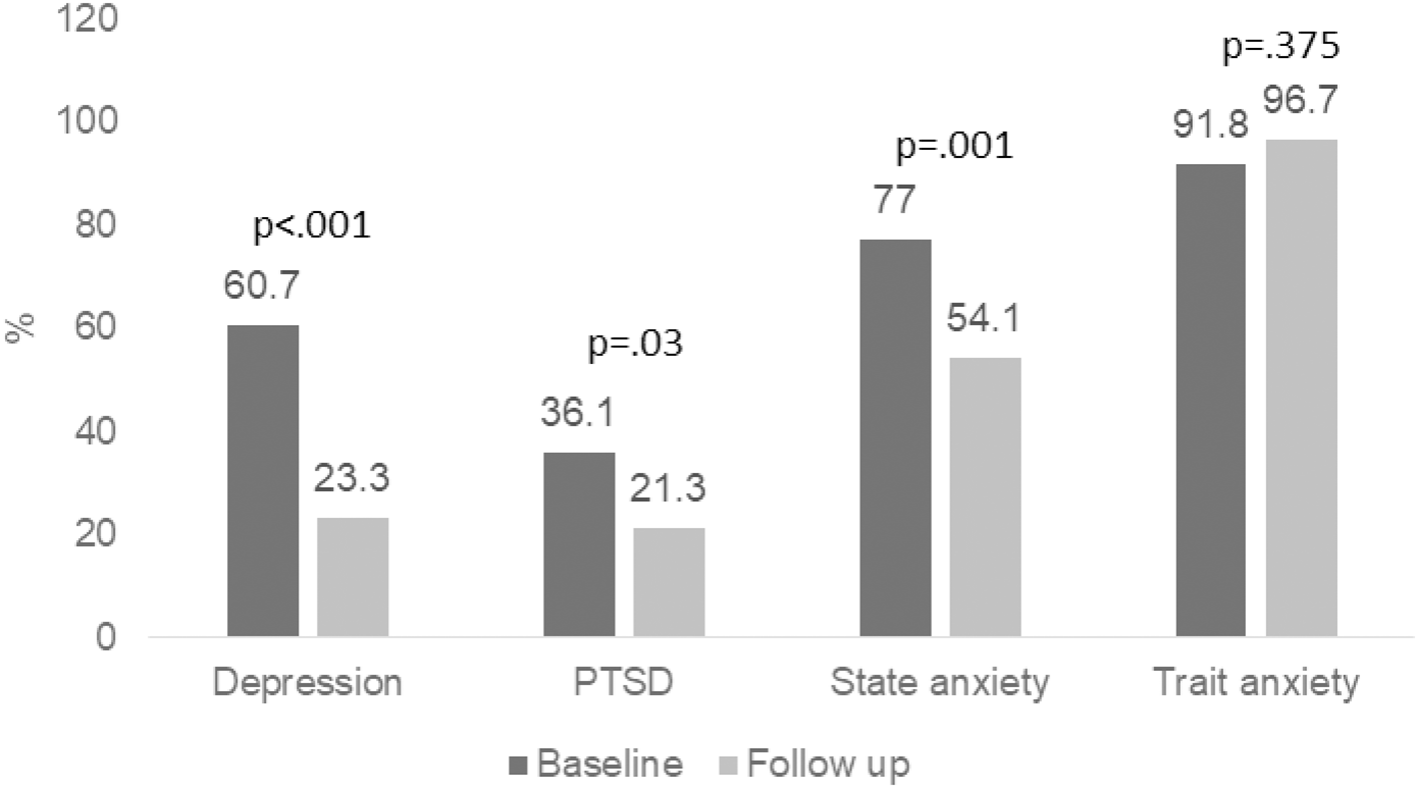
Outcome measures – Percent of patients above clinical cutoff at baseline and follow up. Percentage of patients above clinical cutoff for depression, PTSD, state anxiety and trait anxiety at baseline and follow up. *p* value was calculated using binomial distribution.

For depression, EPDS scores significantly decreased from 14.2 (5.1) at baseline to 10.1 (4.1) at follow-up (t = 7.55; df 59; *p* <.001; Cohen’s *d* = .89). Thirty-seven (60.7%) women were above cutoff for depression at baseline. This number declined to 14 (23.3%) at follow-up (*p* <.0001) (**Figure 1** and **Table 2**).

**Table 2:** Change in symptoms from baseline to follow up (raw scores).

	Mean±SD	CI 95%	t	df	*p*	Effect size
Depression	4.14±4.25	3.04,5.24	7.55	59	<.001*	.89
PTSD	5.07±10.64	2.3,7.8	3.66	58	.001*	.36
State anxiety	6.03±10.36	3.37,8.68	4.54	60	<.001*	.49
Trait anxiety	.55±8.31	-1.58,2.7	0.52	60	0.61	.06

Paired sample t test for depression, PTSD and anxiety symptoms at baseline and follow up. *p≤.001.

Effect size – Cohn’s *d*.

For state anxiety, STAI-S scores decreased from 40.6 (12.6) at baseline to 34.5 (11.8) at follow-up (t = 4.54; df 60; *p* < .001; Cohen’s *d*  = .49). Forty-seven (77.5%) participants were above cutoff for state anxiety at baseline. This number declined to 33 (54.1%) at follow-up (*p = .001*). We did not find significant improvement in trait anxiety scores at follow-up.

PTSS decreased from a mean (SD) PCL-5 score of 31.4 (15.07) at baseline to 26.36 (13.3) at follow-up (t = 3.66; df 58; *p* <.001; Cohen’s *d*  = .36). Twenty-two (36.1%) participants were above cutoff for PTSD at baseline. This number declined to 13 (21.3%) at follow-up (*p* = 0.03).

At baseline, 12 (19.7%) women reported some level of suicidal ideation (any positive answer to question 10 of the EPDS). This number decreased at follow-up to 5 (8.2%) (*p* = .02). The level of suicidal ideation in all five women, was lower in intensity at follow-up compared to their baseline level as indicated by their score to question 10 of the EPDS.

There was no significant change in symptoms of trait anxiety following MBGT. STAI-T scores decreased from 44.3 (9.9) at baseline to 43.6 (8.3) at follow-up (t = .52; df 60; *p* = .61; Cohen’s *d* = .06). Fifty-six (91.8%) participants were above cutoff for state anxiety at baseline. This number showed a nonsignificant increase to 59 (96.7%) at follow-up (*p = .37*).

### Predictors of clinical improvement

We conducted an exploratory correlation analysis between improvement in symptoms of depression, PTSS and anxiety at follow-up and the following sociodemographic characteristics and clinical characteristics: age, number of children, week of stillbirth, time since SB, number of pregnancies, number of deliveries, termination procedure, psychiatric history and treatment with SSRI following SB. The number of living children and deliveries was significantly negatively correlated with improvement in depression and PTSS after MBGT (for depression r(58) = -.28, *p* = .03 and r(58)= -.30, *p* = .02 respectively. For PTSS r(57) = -.25, *p* = .06 and r(57) = -.28, *p* = .03 respectively. Treatment with SSRI following SB and time since SB significantly correlated with improvement in PTSS, r(57) = .31, *p* = .02, and r(57) = .27, *p* = .04 respectively.

To find predictors for clinical improvement after MBGT, we further analyzed our results using multiple linear regression. Multiple linear regression was used to test if number of children, time since SB, use of antidepressants after SB and questionnaire score at baseline significantly predicted improvement in symptoms following MBGT. As expected, higher symptoms severity of depression, PTSS and state anxiety at baseline was associated with greater improvement at follow up (standardized β 0.77, CI (0.52-1.01), *p* < 0.001. standardized β 0.64, CI (0.37-0.91), *p* < 0.001. standardized β 0.56, CI (0.3-0.83), *p* < 0.001. respectively). We also found that use of antidepressants and fewer children were associated with a more robust improvement in depression, (standardized β 0.34, CI (0.1-0.6), *p* = 0.006. standardized β -0.31, CI (-0.52-0.1), *p* = 0.005 respectively) and PTSS (standardized β 0.46, CI (0.19-0.73), *p* < 0.001. standardized β -0.27, CI (-0.49-0.04) *p* = 0.02 respectively) symptom scores following the group intervention (**Table 3**). Time since SB was associated with improvement in PTSS (standardized β 0.3, CI (0.07-0.53), *p* = 0.01), but not with depression or anxiety.

**Table 3:** Predictors for symptom improvement after mind body group therapy (MBGT).

	ΔDepression		ΔPTSS		ΔState Anxiety	
	standardized β (95% CI)	*p*	standardized β (95% CI)	*p*	standardized β (95% CI)	*p*
Medication	0.34 (0.1, 0.6)	0.006	0.46 (0.19, 0.73)	<0.001	0.21 (-0.07, 0.5)	0.14
Number of children	-0.31 (-0.52, -0.1)	0.005	-0.27 (-0.49, -0.04)	0.02	-0.16 (-0.4, 0.08)	0.16
Months since still birth	0.12 (-0.09, 0.33)	0.25	0.3 (0.07, 0.53)	0.01	0.01 (-0.23, 0.25)	0.95
Baseline score	0.77 (0.52, 1.01)	<0.001	0.64 (0.37, 0.91)	<0.001	0.56 (0.3, 0.83)	<0.001

Multiple linear regression analysis for sociodemographic predictors of symptoms improvement after MBGT.

## Discussion

To our knowledge, this is the first study to show the effectiveness of structured MBGT for treating symptoms of depression, anxiety, PTSS and suicidality in women who experienced SB. Women in our cohort presented with these symptoms before participating in MBGT.

As hypothesized, women who experienced SB showed significant improvement in symptoms of depression, PTSS and, state anxiety after participating in MBGT. Improvement in depression and PTSS after MBGT were associated with treatment with SSRI and negatively associated with the number of living children. Time since SB was associated with improvement in PTSS. SB is a known risk factor for suicide (4). In our cohort, suicidal ideation decreased at follow-up suggesting that MBGT may reduce suicide risk in this population.

We used the STAI questionnaire to measure anxiety symptoms. The STAI questionnaire measures trait anxiety, which is considered stable over time, and state anxiety, which is affected by stressful situations such as experiencing SB. We found significant reduction in state anxiety symptoms at follow-up but as expected, did not find a significant change in trait anxiety at follow-up.

Taken together our results show that women who experience SB, benefit from MBGT and show a significant reduction in symptoms intensity after MBGT. As this is a group therapy protocol it represents a possible benefit of peer support as well as a cost-effective approach to treat women who experienced SB.

We examined whether various demographic factors are linked to improvement in depression, anxiety, and PTSS at follow-up. Perhaps unsurprisingly, we found an association between improvement in depression symptoms and PTSS and treatment with SSRIs after SB. It is important to consider that women receiving SSRIs after SB were deemed by a psychiatrist to suffer from severe enough symptoms, requiring treatment with medication. A negative association was found between depression and PTSS improvement and the number of living children at home. This is a surprising finding as it is sometimes assumed by mental health professionals and laypersons that if a woman had a living child at home the psychological impact of SB would be reduced. This finding may suggest that a woman after SB returns home to her children and is often required to function as a parent, diminishing the time she has to process and heal from the traumatic experience. Additionally, some mothers in our MBGT report grief at knowing what they have lost and feelings of guilt towards their living children who were anticipating a sibling. These mothers may require further mental help.

Time since SB was only associated with improvement in PTSS. This perhaps can be explained by the finding that PTSS appear to be highest in the immediate postnatal period, followed by decline as time passes (7, 8, 11).

It is important to note several limitations of this study. First is the small sample size of 61 participants. Future research with larger and more diverse cohorts is necessary to validate the findings and enhance the generalizability of our results. While all women who experienced SB were offered to participate in MBGT, data on those who declined to participate is unavailable. Exploring the sociodemographic characteristics and severity of symptoms of women who declined to participate could provide valuable insights. Furthermore, the absence of a control group prevents the ability to distinguish spontaneous symptom improvement, including the effects of time, from the potential contribution of MBGT. Although time since SB appeared to influence PTSS improvement alone, the observed symptom reduction may reflect a combination of factors including the natural passage of time over the course of the study and at least in part, the participation in MBGT.

Additional studies are required to better define the population that may benefit from MBGT after SB. It would be of interest to compare MBGT for SB to other group and individual intervention protocols. Examining the effect MBGT intervention on women’s family is important and future studies should also address their partner’s and children’s wellbeing. It is of interest to examine mental health outcomes following future pregnancies for women who were treated with MBGT after SB. However, this study is a commendable effort that offers initial insights into the effectiveness of MBGT for women experiencing stillbirth.

## Data Availability

The raw data supporting the conclusions of this article will be made available by the authors, without undue reservation.
